# Comparison of questionnaire responses with biomarkers of tobacco smoke exposure in a Canadian birth cohort at three months of age

**DOI:** 10.1186/1710-1492-10-S1-A60

**Published:** 2014-03-03

**Authors:** Kathleen McLean, Bruce Lanphear, Amanda J Wheeler, Jeff Brook, James Scott, Ryan Allen, Michael Brauer, Malcolm Sears, Padmaja Subbarao, Stuart Turvey, Allan Becker, Piush Mandhane, Tim Takaro

**Affiliations:** 1 Faculty of Health Sciences, Simon Fraser University, Burnaby, Canada; 2 Air Health Science Division, Health Canada, Ottawa, Canada; 3 Air Quality Research Division, Environment Canada, Toronto, Canada; 4 Dalla Lana School of Public Health, University of Toronto, Toronto, Canada; 5 School of Population and Public Health, University of British Columbia, Vancouver, Canada; 6 Department of Medicine, McMaster University, Hamilton, Canada; 7 Department of Pediatrics, Hospital for Sick Children, Toronto, Canada; 8 Department of Pediatrics, University of British Columbia, Vancouver, Canada; 9 Department of Pediatrics & Child Health, University of Manitoba, Winnipeg, Canada; 10 Faculty of Medicine and Dentistry, University of Alberta, Edmonton, Canada

## Background

Exposure to tobacco smoke increases the risk for several adverse health effects in children including wheeze, asthma, and asthma exacerbation [1,2]. Accurately assessing tobacco smoke exposure is important for understanding and preventing these health effects. Questionnaires are a flexible and relatively inexpensive method of assessing exposure, but biomarkers of tobacco smoke exposure are considered more accurate. We developed questionnaire-based exposure models predicting urinary levels of biomarkers cotinine and *trans*-3’-hydroxycotinine (3HC) (metabolites of nicotine) in 3-month old infants using parent-reported questionnaire responses about tobacco smoke exposure from the Canadian Healthy Infant Longitudinal Development (CHILD) Study.

## Methods

We used a manual model building process to build multiple linear regression models predicting urinary concentrations of cotinine, 3HC, and the sum of cotinine and 3HC on a molar basis (Cot+3HC) for 987, 1003, and 983 infants, respectively. Questions were included on the infant’s exposure assessed at 3 months of age and tobacco smoke odour in the home. We also included questions on maternal smoking status and history, passive exposure, and family socio-economic status assessed during pregnancy, as potential indirect measures of the infant’s exposure at 3 months. Adjusted R^2^ values were maximized in the final models.

## Results

During pregnancy, the prevalence of maternal smoking was 2.4 %, and 115 (11.4 %) mothers reported smoking by at least 1 person at home. Of the 144 (14.3 %) infants whose mothers reported that smoking occurred at home when their child was 3 months, 129 (89.6%) and 136 (94.4%) had cotinine and 3HC levels above the detection limit (0.03 ng/mL), respectively. Of the 811 infants who had no parent-reported exposure at 3 months, 538 (66.3%) and 715 (88.2%) had detectable cotinine and 3HC levels, respectively. After correcting for urine dilution, the geometric mean levels were 0.085 ng/mL for cotinine, 0.20 ng/mL for 3HC, and 1.62 picomole/mL for Cot+3HC. The final questionnaire models explained 43.4%, 41.0%, and 42.9% of the variance in cotinine, 3HC, and Cot+3HC levels, respectively.

## Conclusions

Our results indicate that exposure of these infants to tobacco smoke is not completely captured by questionnaires, suggesting that exposure assessment could be improved by using a combination of biomarker and questionnaire methods. Though more detectable, the inclusion of 3HC did not increase the ability of the questionnaires to explain variance in metabolite levels, but 3HC may be important since the ratio of 3HC to cotinine can be used to quantify the rate of nicotine metabolism and variation within populations [3,4].

## References

[B1] Committee on the Assessment of Asthma and Indoor Air Division of Health Promotion and Disease Prevention Institute of Medicine Clearing the Air: Asthma and Indoor Air Exposures 2000 Washington, DC: National Academy Press

[B2] U.S. Department of Health and Human Services The Health Consequences of Involuntary Exposure to Tobacco Smoke A Report of the Surgeon General 2006 Atlanta, GA: U.S. Department of Health and Human Services, Centers for Disease Control and Prevention, Coordinating Center for Health Promotion, National Center for Chronic Disease Prevention and Health Promotion, Office on Smoking and Health [Publications and Reports of the Surgeon General]

[B3] Dempsey D Tutka P Jacob P 3rd Allen F Schoedel K Tyndale RF Benowitz NL Nicotine metabolite ratio as an index of cytochrome P450 2A6 metabolic activity Clin Pharmacol Ther 2004 10 64 72 15229465 10.1016/j.clpt.2004.02.011

[B4] Johnstone E Benowitz N Cargill A Jacob R Hinks L Day I Murphy M Walton R Determinants of the rate of nicotine metabolism and effects on smoking behavior Clin Pharmacol Ther 2006 10 319 330 17015050 10.1016/j.clpt.2006.06.011

